# Regression and machine learning approaches identify potential risk factors for glioblastoma multiforme

**DOI:** 10.1093/braincomms/fcaf187

**Published:** 2025-05-27

**Authors:** Alessio Felici, Giulia Peduzzi, Roberto Pellungrini, Daniele Campa, Federico Canzian

**Affiliations:** Department of Biology, University of Pisa, Pisa 56126, Italy; Genomic Epidemiology Group, German Cancer Research Center (DKFZ), Heidelberg 69120, Germany; Department of Biology, University of Pisa, Pisa 56126, Italy; Classe di Scienze, Scuola Normale Superiore, Pisa 56126, Italy; Department of Biology, University of Pisa, Pisa 56126, Italy; Genomic Epidemiology Group, German Cancer Research Center (DKFZ), Heidelberg 69120, Germany

**Keywords:** Glioblastoma multiforme, machine learning, epidemiology, IGF1, genomics

## Abstract

Glioblastoma multiforme is a lethal disease, with a 5-year survival rate of <10%. The identification of risk factors for glioblastoma multiforme is essential for the understanding of this disease and could facilitate more effective stratification of high-risk individuals. However, our current knowledge of glioblastoma multiforme risk factors is limited. Given the complexity and heterogeneity of the disease, traditional epidemiological approaches may be insufficient to study risk factors for glioblastoma multiforme. The combination of traditional approaches with machine learning models could prove effective in identifying relevant factors for glioblastoma multiforme risk. In this study, we developed glioblastoma multiformerisk models in the UK Biobank cohort using 576 glioblastoma multiforme cases and 302 602 controls. First, 369 exposures were tested with traditional regression models in a case–control study and significant associations were identified. Subsequently, significant features were filtered based on their completion rate and correlation. The selected exposures were then used to develop two machine learning models: a support vector machine and a Multi-Layer Perceptron. To address the imbalance within the subpopulation, two controls per case with full data were selected, resulting in 442 glioblastoma multiforme cases and 884 controls being analysed with the machine learning models. Relevant factors for glioblastoma multiforme risk were identified by explaining the results of the two models with Shapley Additive explanations. Traditional regression methods identified 38 significant associations between environmental exposures and glioblastoma multiforme risk under the Bonferroni threshold (*P* < 1.35 × 10^−4^). Subsequent filtration results in the selection of 12 exposures, which were then analysed with age, sex and a polygenic score using the two machine learning models. Support vector machine and the multi-layer perceptron demonstrated a good sensitivity (0.91 and 0.82, respectively). In addition to age and genetics, Shapley Additive explanations demonstrated significant contributions of insulin-like growth factor 1 blood levels and the right-hand grip strength on the predictions made by the models, with the latter effect potentially being confounded by endogenous testosterone levels. The integration of machine learning with traditional models has the potential to enhance the identification of risk factors for glioblastoma multiforme.

## Teaser

Felici et al. provide novel epidemiological insights by reporting candidate risk factors for glioblastoma multiforme. A two-step approach integrating regression and explainable machine learning models allowed the identification of the insulin-like growth factor 1 and hand-grip strength as candidate risk factors for the disease.

## Graphical Abstract

**Graphical Abstract:**
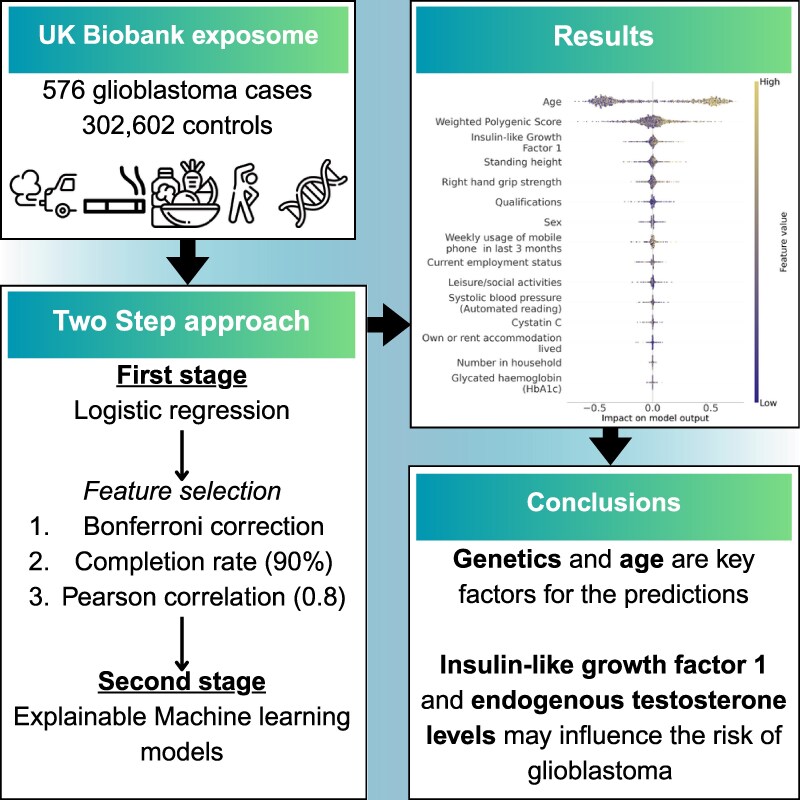

## Introduction

Glioblastoma multiforme (GBM) is the most frequent type of brain neoplasm in adults, accounting for almost 50% of all brain malignancies.^1^ This high-grade glioma occurs more often in males than in females and has a very poor prognosis, with an average 5-year survival rate of <10%.^1,2^ Many efforts have been made to identify risk factors for GBM, but the results are still inconclusive.^3,4^ The risk of GBM increases with age, peaking around the age of 80/85 and then declining.^3,5^ In addition to environmental risk factors, the genetics underlying GBM remain poorly understood. However, recent Genome-Wide Association Studies (GWASs) have begun to provide insights into the genetic architecture of GBM.^6,7^ Currently, GBM is an incurable disease and only a few treatments (such as chemotherapy and surgery) are available, but their effectiveness is extremely limited.^8^ New strategies for studying the aetiology of this disease are being considered, such as the application of machine learning (ML) models.^9^ ML models could integrate traditional approaches to successfully identify relevant factors associated with a specific disease.^10^ The combination of traditional approaches with ML already showed promising results.^10,11^ Following this strategy, we employed a two-stage approach in the UK Biobank (UKBB) cohort. First, we investigated the association between several exposures and germline genetics with GBM risk using traditional approaches based on regression models. Second, with the objective of identifying novel relevant factors and markers for GBM risk, we computed and explained the results of two ML models, namely support vector machine (SVM) and multi-layer perceptron (MLP).

## Materials and methods

### Study subjects

In the UKBB more than 500 000 individuals aged between 37 and 70 years old were recruited between 2006 and 2010. All participants provided informed written consent before their enrolment in the UKBB cohort. Each participant underwent assessment procedures based on self-completed questionnaires, physical measurements, interviews and provided biological samples. Genome-wide single nucleotide polymorphisms (SNP) genotyping was also conducted for 488 377 study participants with two arrays: the Applied Biosystems UK BiLEVE Axiom Array by Affymetrix was used for a subset of 49 950 subjects, while the Applied Biosystems UK Biobank Axiom Array was utilized for the remaining individuals. Following genotyping, standard quality control procedures (i.e. principal component analysis, heterozygosity, missing rate, sex discrepancies and relatedness) and genotype imputation were performed. All the above steps were performed centrally by UKBB. The full description of the genotyping process in UKBB is provided elsewhere.^12^ Data used in this study were obtained from UKBB under project ID 66 591. The UKBB study was approved by the North-West Multi-centre Research Ethics Committee (MREC, REC reference number: 21/NW/0157) and this study complies with the Declaration of Helsinki. This study adhered to the Strengthening the Reporting of Observational Studies in Epidemiology (STROBE) statement for case–control studies.

### Ascertainment of cases and controls

The present study was designed as a case–control study. From the overall 502 420 participants, 472 622 individuals with a White, British, Irish, or any other European ancestry background (UKBB field 2100 codes 1, 1001, 1002, and 1003, respectively) were selected. GBM cases were selected using cancer registry data. ICD10 codes identifying malignant neoplasms of the brain (C71.9) were retrieved using the UKBB data field 40006 (‘type of cancer’). The ICD10 codes were then mapped to ICD11 codes indicating primary malignant neoplasms of brain of unknown or unspecified type (2A00.5) using the WHO ICD10/ICD11 converting map (https://icd.who.int/browse/2025-01/mms/en, ‘Info’ tab). The histological codes for GBM were identified via the International Classification of Diseases (ICD-O-3). GBM NOS (i.e. Not Otherwise Specified) cases were identified by combining the GBM ICD11 code with the relative histological code (9440), which was retrieved for each case from the UKBB data field 40011 (‘histology of cancer tumour’). Giant cell glioblastoma (histology code 9441) and gliosarcoma (9442) were excluded from the analysis, as they represent distinct histological variants of GBM. Finally, using the UKBB data field 40012 (‘behaviour of cancer tumour’), only GBM cases that arose in a primary site (Code 3) or with a microinvasive behaviour (Code 5) were used. Controls were identified as all participants without a cancer diagnosis in self-reported (UKBB Data Field 20001), hospital inpatient (UKBB Data Field 41270), or cancer registry (UKBB Data Field 40006) data fields. Following the exclusion of all participants who had withdrawn their consent, a total of 576 GBM cases and 302 602 controls were finally selected for analysis. Details on the study population are reported in Table 1.

**Table 1:** Description of the study population

Group	Cases	Controls	Total	*P*-value
Females				
*N*	221	161 026	161 247	2.11 × 10^−99^
Mean age [SD]	65.25 [8.13]	55.80 [8.00]	55.81 [8.01]	
Males				
*N*	355	141 576	141 931	1.83 ×10^−57^
Mean age [SD]	65.77 [7.77]	55.39 [8.19]	55.42 [8.20]	
Total				
*N*	576	302 602	303 178	3.66 × 10^−154^
Mean age [SD]	65.58 [7.92]	55.61 [8.09]	55.63 [8.10]	

Number of GBM cases and controls in the UK Biobank study population, divided by sex. *P*-values refer to the comparison between ages for cases and controls using the Mann–Whitney U-test.

### Exposome analysis

A total of 369 exposures belonging to 41 categories (hereafter referred to as the exposome) were tested for association with GBM risk. A detailed list of the tested exposures along with their respective measurement unit, number of cases and controls available for analysis and completion rate is provided as Supplementary Table 1. All these exposures were tested individually for association with GBM risk. A total of 26 exposures were derived from existing variables in the UKBB set and their description is provided in Supplementary material.

### *Post hoc* analyses

Further exploratory analyses were conducted to better understand the observed association between hand-grip strength and GBM risk. This decision is contingent upon the evidences linking hand-grip strength to cognition and grey matter volume.^13,14^ Specifically, sensitivity analyses were conducted to investigate the potential underlying the role of testosterone in this observed association. These *post hoc* analyses were conducted by stratifying the association between right-hand grip strength and GBM risk according to tertile of testosterone levels computed based on the distribution of the levels of this hormone in controls. Additionally, sensitivity analyses were also performed for the left and overall hand-grip strength. Testosterone effects may also drive the observed associations between whole body fat-free and whole body water mass with GBM risk. Indeed, a positive association between testosterone levels and both increased lean mass and strength has been found,^15-17^ while lower levels of this hormone have been associated with an increase in abdominal fat accumulation.^18^ Therefore, *post hoc* sensitivity analyses were performed also for these observed associations.

### Polygenic score

Genetic susceptibility to GBM was tested by computing a polygenic score (PGS). To compute the PGS, 13 SNPs were selected from the largest GWAS meta-analysis available,^7^ retrieved via GWAS catalog.^19^ Linkage disequilibrium pruning was performed on LDlink^20^ using the European population (excluding individuals with Finnish ancestry), with *r*^2^ < 0.2 and minor allele frequency (MAF) = 0.05 as thresholds. SNPs were also filtered for reported *P*-value of association with GBM risk, and only those SNPs with a *P*-value lower than 5 × 10^−8^ were retained. Eleven SNPs were finally selected to compute the PGS.

The GBM PGS was calculated as a weighted measure by summing up the product of the number of risk alleles (0, 1 or 2) multiplied by the effect reported in the original publication for each SNP,^7^ using the following formula:


$$\mathrm{PGS}=\overset{n}{\underset{\;j=1}{\sum }}\;X_{ij}\beta_{j}.$$

where *n* represents the number of SNPs used to calculate the GBM PGS, *X_ij_* represents the number of risk alleles harboured from the participant *i* for the SNP*_j_* and *β* is the reported effect for SNP*_j_*. The PGS was tested for association with the outcome as a continuous exposure and ranked in tertiles calculated on the distribution in the controls. A list of the selected SNPs, chromosome position, effect allele (A1), MAF, effect [odds ratios (OR)] and *P*-value is reported as Supplementary material.

### ML models

With the aim of predicting GBM cases, a SVM^21^ and a MLP^22^ were employed in this study. These models were subsequently explained with the aim of identifying relevant predictors associated with an increased risk of GBM. Prior to the deployment of the models, a series of different steps were undertaken, as detailed in the following paragraphs.

### Feature selection and pre-processing

Sixteen features which were significant under the Bonferroni threshold (*P* = 0.05/369 = 1.355 × 10^−4^) and with a complete rate equal to or >90% were identified for feature selection. Due to the constraints imposed by the missing values when providing data to ML models, it was necessary to exclude the female-specific exposure ‘number of live births’ (since no males would have remained after the removal of missing data), and the ‘age stopped smoking’ exposure (since it is only applicable to former smokers). Consequently, 14 exposures remained. Then, three correlation analyses were conducted, employing Spearman correlation, Pearson correlation or Cramer's V matrix for continuous, ordinal and categorical exposures, respectively. For the correlation analysis, a threshold of *ρ* ≤ 0.8, *r* ≤ 0.8 and V ≤ 0.8 were used, respectively. A correlation was identified between ‘whole body fat-free mass’, ‘whole body water mass’ and ‘standing height’ (*ρ* = 0.82 for both). Considering also the perfect positive correlation between ‘whole body fat-free mass’ and ‘whole body water mass’ (*ρ* = 1.00), these two measures were discarded and only ‘standing height’ was retained for further analysis. Along with age, sex and the PGS, 12 exposures were ultimately selected. The selected exposures, along with their respective data type and measurement unit, number of cases and controls and completion rate are presented in Table 2. Continuous exposures were normalized using *z*-scores. Conversely, categorical exposures were encoded using the one-hot encoding method, thereby creating a binary column for each category (i.e. dummy variables). Finally, tertile exposures initially computed for glycated haemoglobin HbA1c, IGF-1 and cystatin C were used as the original continuous exposures.

**Table 2:** Selected features to be used for GBM risk prediction model computation

Exposure	UKBB field	Type	Unit	Controls	Cases	CR
Standing height	50	Continuous	cm	301 570	574	99.66
Cystatin C	30 720	Continuous	mg/L	283982	533	93.84
Glycated haemoglobin (HbA1c)	30 750	Continuous	mmol/mol	282 881	534	93.48
IGF1	30 770	Continuous	nmol/L	282 511	528	93.36
Systolic blood pressure (automated reading)	4080	Continuous	mmHg	281 148	531	92.91
Right-hand grip strength	47	Continuous	Kg	301 013	575	99.48
Current employment status	6142	Categorical		301 696	576	99.70
Qualifications	6138	Categorical		297 928	561	98.45
Own or rent accommodation lived	680	Categorical		298 755	567	98.73
Number in household	709	Continuous	people	301 029	571	99.48
Leisure/social activities	6160	Categorical		301 796	573	99.73
Weekly usage of mobile phone in last 3 months	1120	Categorical		294 683	565	97.38

The table reports the 12 features selected for the computation of the GBM risk prediction model, in addition to age, sex and the PGS. The 12 selected features are all the exposures that resulted associated with GBM risk in the association analysis, after accounting for multiple comparisons, and with a completion rate (CR) ≥ 90%.

### Training and test sets

Given the considerable imbalance between the number of cases and controls in our dataset, we employed an under-sampling technique to randomly select only two controls per case. The under-sampling of the majority class (i.e. the controls) was repeated 1000 times to identify the optimal combination of cases and controls. Following the removal of all participants with missing values, 442 GBM cases and 884 controls remained and were used to train the models. Subsequently, the dataset was divided, with the initial 80% used for the actual training process of the models and the remaining 20% employed for testing.

### Hyperparameter tuning

Bayesian optimization was used to tune the hyperparameters of the two models. Briefly, Bayesian optimization uses a probabilistic model and an acquisition function to select the best hyperparameter combination. In this study, the Bayesian Optimization ‘bayopt’ approach^23^ was employed. Details on the selected hyperparameters are reported as Supplementary material.

### Performance evaluation

Considering that the accuracy may be less informative when working with imbalanced data due to the presence of a higher number of controls than cases,^24^ the performance of the models was evaluated relying on sensitivity and F1-score.


$$\mathrm{Sensitivity}=\frac{\mathrm{True}\;\mathrm{positives}}{\mathrm{True}\;\mathrm{positives}+\mathrm{False}\;\mathrm{negatives}},$$

$$F1=\frac{2\times \mathrm{precision}\times \mathrm{sensitivity}}{\mathrm{precision}+\mathrm{sensitivity}}.$$

Other performance metrics were also calculated, including accuracy, the area under the receiver operating characteristic curve (AU-ROC curve), the precision-recall curve (PRC) and specificity:


$$\mathrm{Accuracy}=\frac{\mathrm{True}\;\mathrm{positives}+\mathrm{True}\;\mathrm{negatives}}{\mathrm{True}\;\mathrm{Positives}+\mathrm{False}\;\mathrm{positives}+\mathrm{True}\;\mathrm{negatives}+\mathrm{False}\;\mathrm{negatives},}$$

$$\mathrm{Specificity}=\frac{\mathrm{True}\;\mathrm{negatives}}{\mathrm{False}\;\mathrm{positives}+\mathrm{True}\;\mathrm{negatives}}.$$

### ML models explanation

Finally, SVM and MLP outputs were explained using Shapley Additive Explanations (SHAP).^25^ SHAP is an explainable AI method that is used to assess the impact of each feature on each model's output. To graphically represent the explanations, a beeswarm summary plot was generated. A SHAP beeswarm plot facilitates the visualization of the impact of each feature on the output generated by the model.

### Statistical analysis

For the association between each exposome variable, the PGS and GBM risk, OR and their 95% confidence intervals (95% CI) were calculated using multivariable logistic regression models, adjusted for age and sex when necessary. All logistic models were performed in RStudio, version 4.2.2. The significance of each association for the association analysis was evaluated using a Bonferroni-corrected *P*-value threshold of 1.355 × 10^−4^ (0.05/369). Model development and data preparation procedures were performed in Python v.3.9.13, using Spyder IDE v.5.2.2.

## Results

### The effect of the exposome on GBM risk

After accounting for multiple comparisons, 38 associations were found to be significant under the Bonferroni-corrected *P*-value threshold. Among these associations, seven had never been previously reported for GBM risk. Specifically, we observed increased GBM risk for higher values of right-hand grip strength (HGS) (OR = 1.03, 95%CI = 1.02–1.04, *P* = 4.30 × 10^−9^ per 1 Kg increase), whole body fat-free mass (OR = 1.03, 95%CI = 1.02–1.04, *P* = 1.02 × 10^−5^ per 1 Kg increase) and whole body water mass (OR = 1.04, 95%CI = 1.02–1.06, *P* = 1.93 × 10^−5^ per 1 Kg increase). Moreover, reduced GBM risk was observed for high cystatin C levels when comparing tertile T3 with T1 (OR = 0.58, 95%CI = 0.46–0.72, *P* = 1.44 × 10^−6^), for older age at first sexual intercourse (OR = 0.95, 95%CI = 0.93–0.97, *P* = 3.51 × 10^−5^ per 1 year increase) and for attending (once a week or more often) a religious group, compared to attending any other group activity (OR = 0.48, 95%CI = 0.33–0.69, *P* = 6.53 × 10^−5^). Finally, we observed a significant and inverse association between high sex-hormone binding globulin (SHBG) levels and GBM risk (OR = 0.60, 95%CI = 0.47–0.76, *P* = 1.83 × 10^−5^ for T3 versus reference T1). Regarding the exposures more extensively reported in the literature in association with GBM, the associations between alcohol consumption, body mass index (BMI) and aspirin with GBM risk were all non-significant under the Bonferroni threshold.

**Figure 1:**
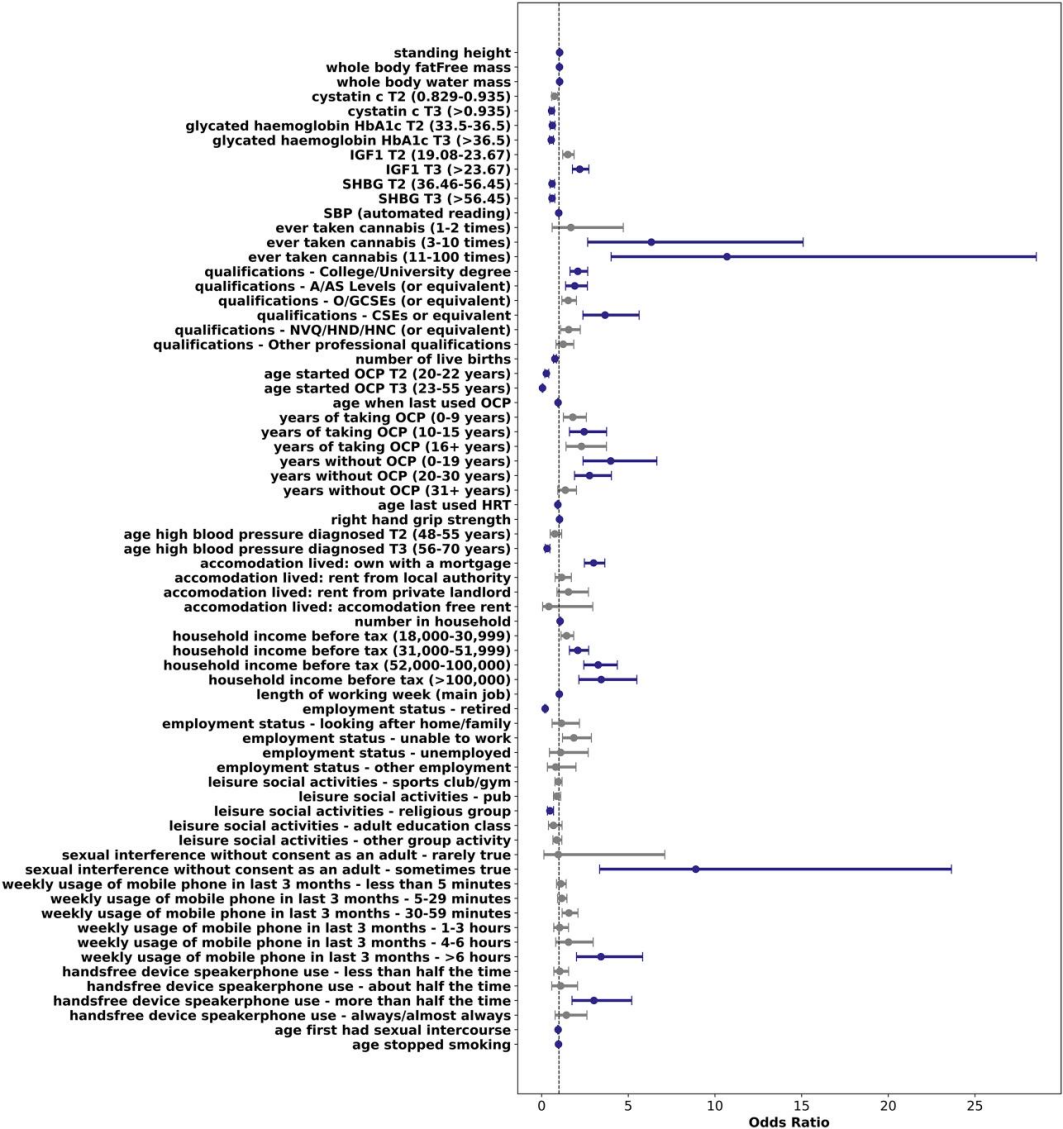
**Significative association with GBM risk under the Bonferroni-corrected *P*-value (*P* = 1.355 × 10^−4^).** Odds ratio and their relative CI for the association between each exposure and GBM risk are computed using logistic regression models on 576 cases and 302 602 controls and are reported on the *x*-axis. ‘Ever taken cannabis—>100 times’, ‘employment status—unpaid or voluntary work’, ‘current employment status—full or part-time student’, ‘sexual interference without consent as an adult (by partner/ex-partner)–often’, ‘sexual interference without consent as an adult (by partner/ex-partner)—very often’ and ‘accommodation lived—care home’ were removed from the forest plots due to the absence of cases. In the forest plots, categorical exposures are reported along with their parental category. A/AS levels, advanced/advanced subsidiary levels; SHBG, sex-hormone binding globulin; SBP, systolic blood pressure; GCSE, general certificate of secondary education; CSE, certificate of secondary education; NVQ, national vocational qualification; HND, higher national diploma; HNC, higher national certificate; OCP, oral contraceptive pill; IGF1, insulin-like growth factor 1; T2, Tertile 2; T3, Tertile 3.

In addition, we identified a significant positive association between the weekly usage of mobile phones for more than 6 h in the last 3 months (compared with not using it at all) and GBM risk (OR = 3.42, 95%CI = 2.01–5.83, *P* = 5.69 × 10^−6^). Furthermore, a positive and significant association was observed between the highest concentration of IGF-1 blood levels (tertile T3) and GBM risk (OR = 2.20, 95%CI = 1.78–2.73, *P* = 5.34 × 10^−1^. All the associations between exposome exposures and GBM risk are reported as Supplementary Table 2, while Bonferroni-significant associations (along with their respective categories, when the variables were analysed on a non-continuous scale) are reported in Fig. 1.

### *Post hoc* analyses

A significant association was observed between right, left and overall hand-grip strength and GBM risk among individuals with medium (tertile T2) and high (tertile T3) testosterone levels. These associations were not significant among individuals with the lowest levels of testosterone (tertile T1), thus suggesting a possible modulatory effect exerted by testosterone levels. We observed a similar trend also for whole body fat-free mass and whole body water mass, but in this case the strongest association with GBM risk was observed, for both, in the intermediate strata (i.e. tertile 2). Results for *post hoc* analyses are reported in Table 3.

**Table 3:** Sensitivity analyses based on testosterone levels for the association between hand-grip strength, whole body fat-free and water masses and GBM risk

Exposure	Testosterone	Cases	Controls	OR	95%CI	*P*-value
Whole body fat-free mass	T1	109	86 314	1.01	[0.97–1.05]	5.49E–01
Whole body fat-free mass	T2	188	86 310	1.03	[1.01–1.05]	6.73E–03
Whole body fat-free mass	T3	191	86 304	1.02	[1.04–1.05]	2.08E–02
Whole body water mass	T1	109	86 314	1.02	[0.96–1.07]	5.85E–01
Whole body water mass	T2	188	86 310	1.04	[1.01–1.07]	7.56E–03
Whole body water mass	T3	191	86 304	1.03	[1.03–1.06]	2.92E–-02
Right HGS	T1	109	86 314	1.01	[0.98–1.04]	5.66E–01
Right HGS	T2	188	86 310	1.04	[1.02–1.05]	2.22E–04
Right HGS	T3	191	86 304	1.04	[1.02–1.06]	1.65E–05
Left HGS	T1	109	86 314	0.998	[0.97–1.03]	9.35E–01
Left HGS	T2	188	86 310	1.04	[1.02–1.06]	1.60E–05
Left HGS	T3	191	86 304	1.05	[1.03–1.06]	8.03E–07
HGS	T1	109	86 314	1.005	[0.97–1.04]	7.95E–01
HGS	T2	188	86 310	1.04	[1.02–1.06]	2.32E–05
HGS	T3	191	86 304	1.05	[1.03–1.07]	1.15E–06

HGS, hand-grip strength.

### GBM risk and genetics

The weighted PGS was strongly associated with GBM risk in both continuous (OR = 1.17, 95%CI = 1.14–1.19, *P* = 7.81 × 10^−44^ per 1 PGS unit increase) and tertile analysis (for T3 versus T1: OR = 3.47, 95%CI = 2.73–4.40, *P* = 1.97 × 10^−24^). Association results for the PGS are reported in Table 4.

**Table 4:** Association analysis for the computed PGS and GBM risk

PGS analysis	Controls	Cases	OR	95%CI	*P*-value
Weighted PGS	294 356	555	1.17	[1.14–1.19]	7.81 × 10^−44^
Weighted PGS T3—T1	98 191	87	ref	ref	ref
Weighted PGS T3—T2	98 575	160	1.84	[1.42–2.40]	4.58 × 10^−06^
Weighted PGS T3—T3	97 590	308	3.47	[2.73–4.40]	1.97 × 10^−24^

### ML risk factors analysis and risk prediction

**Figure 2:**
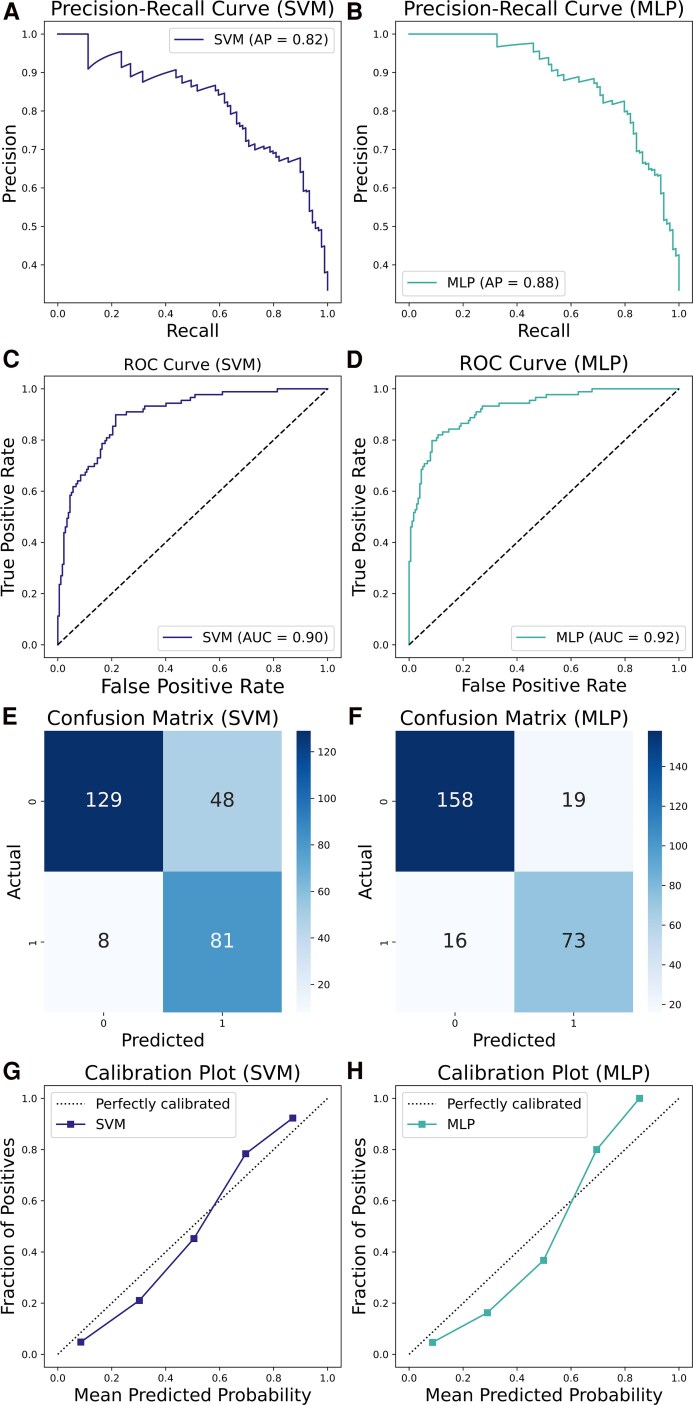
**Overview of the performance of the SVM and MLP models.** Precision-recall curves (summarized as average precision), AUC curves, confusion matrices and calibration plots for the SVM (**A**, **C**, **E**, **G**, respectively) and the MLP (**B**, **D**, **F**, **H**, respectively) models. Results are referred to a subsample of 442 cases and 884 controls with complete rate >90%.

Both models exhibited satisfactory generalization ability, achieving a good degree of accuracy (SVM = 0.79, MLP = 0.87) and a high sensitivity (SVM = 0.91, MLP = 0.82). Nevertheless, the models exhibited a relatively low precision on cases, indicating a notable presence of false positives. Additionally, the MLP model yielded a better specificity (MLP = 0.89, SVM = 0.73). The resulting F1-scores on cases were generally satisfactory (SVM = 0.74, MLP = 0.81). Additionally, the models demonstrated higher classification metrics on controls than in cases. Finally, the SVM and the MLP developed in this study exhibited an optimal trade-off between precision and recall (PRC = 0.82 and 0.88, respectively) and demonstrated a notable capacity for class discrimination [area under the curve (AUC) = 0.90 for SVM and AUC = 0.92 for MLP]. Figure 2 illustrates the performance of both models. Metrics for the two models are reported in Supplementary Table 3.

**Figure 3:**
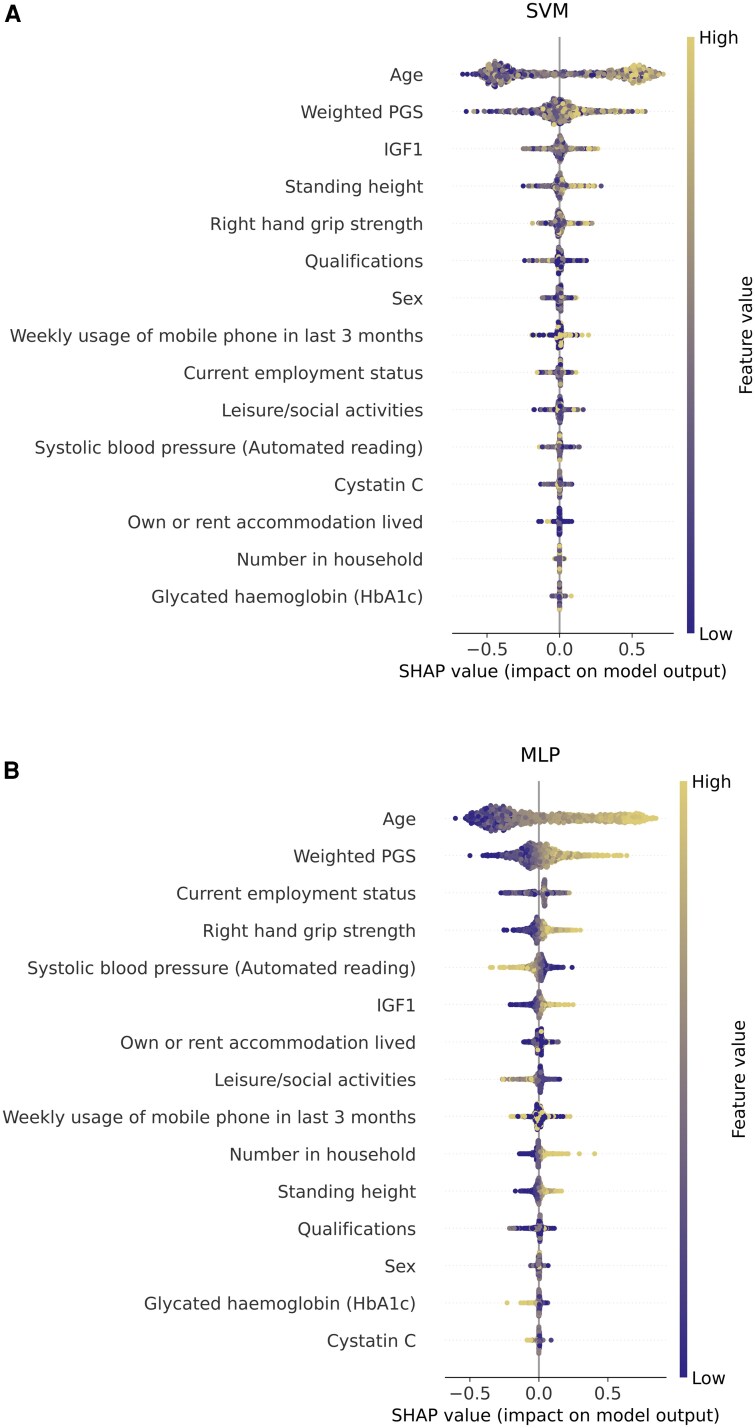
**SHAP explanations.** SHAP explanations for the SVM (**A**) and the MLP (**B**) models. The impact of each feature on models’ performance is reported as SHAP values in the beeswarm summary plot. Within a beeswarm summary plot, dots represent high or low values of a feature, and the SHAP values represent the impact that each feature has on the model's decisional output, with positive or negative SHAP values representing a positive or negative impact of a feature on the model's decisional output, respectively. Results are referred to a subsample of 442 cases and 884 controls with complete rate >90%. SVM, support vector machine; MLP, multi-layer perceptron; IGF1, insulin-like growth factor 1; PGS, polygenic score.

When looking at the SHAP values, the most important features were age and the PGS value of each participant. A taller height, high IGF-1 blood levels and a high right HGS were the most important features for SVM predictions. On the other hand, elevated right HGS and IGF-1 blood levels, employment status and low systolic blood pressure were identified as significant contributors to the predictions of the MLP model. SHAP explanations for SVM and MLP are reported in Fig. 3. Additional information about the SVM and the MLP models is reported in Supplementary material.

## Discussion

The exposome analysis highlighted seven new associations never reported before for GBM risk. HGS has been already studied in association with several cancer types,^26,27^ with the overall tendency of higher HGS associated with cancer risk reduction. In our study, higher right HGS levels were associated with an increased GBM risk. We hypothesize that this association may be confounded by the sex of the participants due to gender-related differences in HGS.^28^ Moreover, considering the plausible role of testosterone in GBM cell proliferation^29^ and its association with muscle strength,^16^ the reported association between right HGS and GBM risk may be influenced by the endogenous levels of this hormone. Additionally, testosterone levels may also influence the observed association between whole body fat-free mass and whole body water mass.^15-17^ To test these hypotheses, we performed *post hoc* sensitivity analyses. Significant associations between HGS, whole body fat-free mass and whole body water mass and GBM risk were observed for higher levels of testosterone. Nonetheless, besides testosterone, it is possible that other confounders could be involved in the aforementioned associations. Furthermore, our findings indicated an inverse association between high SHBG blood levels and GBM risk in our cohort. This association is biologically plausible, given the proposed adverse effects of testosterone on GBM cell growth and proliferation^29^ and the suppression of the bioavailability of the bioactive form of this hormone operated by SHBG.^30^

Additionally, we found an inverse association between GBM risk and cystatin C levels. The role of cystatin C in GBM has been mostly investigated with immunohistochemical assays and cell cultures,^31,32^ highlighting an increased aggressiveness of GBM in association with reduced cystatin C levels. Cystatin C is an inhibitor of Cathepsin B, a protease which is overexpressed in GBM.^32^ The association observed in this study may be explained by the inhibition of Cathepsin B exerted by cystatin C.

Inverse associations with GBM risk were also observed for a later age at first sexual intercourse, and for attending religious groups when compared with any other group activities. While an earlier sexual debut has been repeatedly associated with mental distress and anxiety-related and depressive disorders,^33,34^ better well-being was observed for religious attendance.^35^ The mental well-being behind these two exposures may therefore imply a better adherence to a healthy lifestyle.^36,37^ Nonetheless, these two associations should be contextualized as they may be confounded by other factors.^38,39^ In particular, the early sexual debut could be a result of the adherence at younger ages to unhealthy lifestyles, such as smoking.^40^ In addition, the adherence to a healthy lifestyle in people attending religious groups could be a consequence of an increased social engagement and support.^35,41^ Finally, both associations could also be confounded by a higher educational attainment^38^ and household socioeconomic status.^35,42^

Finally, in our cohort, increases in whole body fat-free and water masses were positively associated with GBM risk. This association may be confounded by other anthropometric factors related to body composition. In addition, an underlying genetic predisposition overlapping with both the increase in fat-free mass and cancer risk has been suggested elsewhere.^43^

Furthermore, our findings indicate a strong role of genetics in GBM risk. We built a PGS (using the largest available GWAS meta-analysis^7^) which displayed a strong positive association with GBM risk. As expected, in this study, the majority of GBM cases exhibited the highest level of risk alleles (i.e. tertile T3).

Finally, we built an SVM and an MLP model to identify significant predictors of the disease. These models displayed a good trade-off between recall and precision. Nonetheless, the two models displayed a non-perfect calibration, which could be related to the small sample size available for this study. However, the objective of the models was not the prediction of GBM risk. Rather, they were developed to identify relevant predictors associated with an increased risk of the disease. The decisional processes of the SVM and the MLP were explained with SHAP explanations.

For both models, age and high level of PGS were the most important predictors. Although GBM is more frequently diagnosed in males than females, the sex of the participants did not appear to be a significant factor for the classification process of the models developed in this study. In the SVM model, participants with taller height and higher IGF-1 blood levels were more likely to be predicted as cases. IGF-1 blood levels were also an important feature for the MLP model. A taller height has been linked with an increased risk of central nervous system tumors,^44^ and its effect on cancer risk may be partially mediated by IGF-1 levels.^45,46^

In the case of the MLP model, the employment status and the systolic blood pressure were reported among the most important predictors. While it is difficult to ascertain the true impact of the current employment status from the beeswarm summary plot, participants with a lower systolic blood pressure were more likely to be classified as cases. This result is discordant with the reported evidence in the literature^47^ and could be due to cohort-specific structures and unmeasured confounders. Finally, the right HGS was identified as a significant predictor in both the models. This result may be related to sexual-related differences in GBM incidence and testosterone levels which are higher in males than in females, as previously stated.

Our study has several strengths and limitations. We performed our analyses on a GBM sample which was reliable to perform association analysis. We found some associations reported in the literature to be non-significant in our UKBB population, such as alcohol consumption, non-steroidal anti-inflammatory drugs (NSAID) use and BMI. Consequently, we decided not to include them in further analyses and instead we performed a rigorous feature selection to retain only significative features that would not increase the complexity of the models considering the modest sample size remaining after the feature selection process (442 cases). Lastly, we did not have the possibility of replicating the SVM and MLP models and their SHAP explanations in an independent prospective cohort, and this is a main limitation of the current study. It should also be noted that the present study has been conducted exclusively on individuals of European ethnicity. Consequently, the results here obtained may not be generalized to other populations. Finally, given the absence of information regarding molecular subtypes within the UKBB cohort, the selection of GBM cases based on isocitrate dehydrogenase-wildtype as reported by the most recent WHO classification of CNS tumours (5th edition, 2021) was not possible because of the lack of this information in UKBB. Consequently, GBM NOS cases were analysed in this study, a classification permitted by the WHO 2016 classification of central nervous tumors.^48^ In conclusion, we highlighted high IGF-1 blood levels and a low HGS as candidate factors for GBM risk. The aetiology of GBM remains difficult to disentangle. However, ML has the potential to improve the study and the identification of significant risk factors for this disease.

## Supplementary Material

fcaf187_Supplementary_Data

## Data Availability

The data used in this study were accessed through the UK Biobank Resource under application no. 66591. The data underlying this article cannot be shared directly. ‘Bona fide’ researchers can access UK Biobank data by registering and applying on the UK Biobank Platform: http://ukbiobank.ac.uk/register-apply/. The code employed in the construction of the machine learning models used in the present study is available at: https://github.com/alessiohappy/Epidemiologic-insights-into-Glioblastoma.
