# Supplementary material for: Regression and machine learning approaches identify potential risk factors for glioblastoma multiforme
Source: Brain Commun. 2025 May 27;7(3):fcaf187. doi: 10.1093/braincomms/fcaf187 (PMC12127608; doi:10.1093/braincomms/fcaf187)
Supplement: fcaf187_Supplementary_Data [file fcaf187_supplementary_data.zip › Supplementary_material.docx]

**Summary**

1. **Materials and methods**
   1. **Exposome analysis**
   2. **Polygenic score (PGS)**
   3. **Machine learning: hyperparameter tuning**
      1. **SVM model**
      2. **MLP model**
2. **Hyperparameter tuning**
   1. **SVM model**
   2. **MLP model**
3. **SHAP barplot**
4. **References**
5. **Materials and methods**
   1. **Exposome analysis in the UK Biobank cohort: derived exposures**

For the exposome analysis, twenty-six exposures were derived from existing ones in UKBB and then tested for association with GBM risk.

**NLR, PLR, LMR, SII.**

From the category “blood count”, using the UKBB data fields “neutrophil count” (30140), “lymphocyte count” (30120), “monocyte count” (30130), and “platelet count" (30080), the neutrophil-to-lymphocyte ratio (NLR), the platelet-to-lymphocyte ratio (PLR), the lymphocyte-to-monocyte ratio (LMR), and the systemic immune inflammation index (SII) were derived as follows:

$$NLR=\frac{neutrophilcount}{lymphocytecount}PLR=\frac{plateletcount}{lymphocytecount}LMR=\frac{lymphocytecount}{monocytecount}SII=\frac{neutrophilcount*plateletcount}{lymphocytecount}$$

**Blood groups.**

The blood group for each participant was derived according to the haplotype for the polymorphisms rs8176746 and rs687289, as reported by Wolpin and colleagues^1^, since both polymorphisms display a full correlation (r^2^ = 1) with blood groups’ B and O alleles, respectively. Specifically, individuals with the A blood group were identified among those harboring two G alleles for the polymorphism rs8176746, and with one (blood group A0) or two (blood group AA) A alleles for the polymorphism rs687289. Individuals with the B blood group were identified among those harboring two T alleles for the polymorphism rs8176746, and with one (blood group B0) or two (blood group BB) A alleles for the polymorphism rs687289. Individuals with the AB blood group were identified among those with one T allele for the polymorphism rs8176746, and with one or two A alleles for the polymorphism rs687289. Finally, individuals with the blood group 0 (zero) were identified among those without the A allele for the polymorphism rs687289.

| Blood group | rs8176746 haplotype | rs687289 haplotype |
| --- | --- | --- |
| 0 | GG | GG |
|  | TG | GG |
|  | TT | GG |
| A | GG | AG |
|  | GG | AA |
| B | TT | AG |
|  | TT | AA |
| AB | TG | AG |
|  | TG | AA |

**Never eat eggs, dairy products, wheat, sugar.**

Using the UKBB data field 6144 (never eat eggs, dairy, wheat, sugar), four exposures were derived by creating dummy variables:

1. Never eat eggs or food containing eggs: from the original exposure, category 1 was used as 1, while all other categories (2, 3, 4, 5) were used as 0.
2. “Never eat dairy products”: from the original exposure, category 2 was used as 1, while all other categories (1, 3, 4, 5) were used as 0.
3. “Never eat wheat”: from the original exposure, category 3 was used as 1, while all other categories (1, 2, 4, 5) were used as 0.
4. “Never eat sugar”: from the original exposure, category 4 was used as 1, while all other categories (1, 2, 3, 5) were used as 0.

**Had twins.**

Females who delivered twins were identified using the UKBB data field 2744 (birth weight of first child), using the code -2 (“only had twins”).

**Years of taking and years without oral contraceptive pill (OCP).**

For female participants, the “years of taking OCP” was derived as the difference between the age at which the participant started to use the OCP (UKBB data field 2794) and the age when the participant last used the OCP (UKBB data field 2804). Moreover, the “years without OCP” exposure was derived as the difference between the age at recruitment and the UKBB data field 2804 for females who ever used the OCP (identified with the UKBB data field 2784, “ever taken oral contraceptive pill”). Both the exposures were used as categories. Females who reported to never used the OCP were used as the reference category. Categories thresholds are reported in the following table.

| Exposure | Categories | | | |
| --- | --- | --- | --- | --- |
|  | Reference (1) | 2 | 3 | 4 |
| years of taking OCP | No use | 0 – 9 | 10 – 15 | 16+ |
| years without OCP | No use | 0 – 19 | 20 – 30 | 31+ |

**Years of taking and years without hormone replacement therapy (HRT).**

A similar approach as that reported above was used for HRT.
For female participants, the “years of taking HRT” was derived as the difference between the age at which the participant started to use the HRT (UKBB data field 3536) and the age when the participant last used the HRT (UKBB data field 3546). The “years without HRT” exposure was derived as the difference between the age at recruitment and the age last used HRT for females who ever used the HRT (identified with the UKBB data field 2814, “ever used hormone replacement therapy”). Both the exposures were used as categories. Females who reported to never used the HRT were used as the reference category. Categories thresholds are reported in the following table.

| Exposure | Categories | | | | |
| --- | --- | --- | --- | --- | --- |
|  | Reference (1) | 2 | 3 | 4 |  |
| years of taking HRT | No use | 0 – 4 | 5 – 10 | 11+ |  |
| years without HRT | No use | 0 – 4 | 5 – 10 | 11+ |  |

**Years of smoking and years without smoking.**

The “years of smoking” exposure was derived, for former smokers, by the difference between the age when stopped smoking (UKBB data field 2897) and the age when started smoking (UKBB data field 2867). The “years without smoking” exposure was derived, for former smokers, by subtracting the age when started smoking to the age recorded at recruitment. Individuals who reported never to have smoked were used as the reference group for both the exposures. Categories thresholds are reported in the following table.

| Exposure | Categories | | | | |
| --- | --- | --- | --- | --- | --- |
|  | Reference (1) | 2 | 3 | 4 |  |
| years without smoking | No use | 0 – 9 | 10 – 25 | 26+ |  |
| years of smoking | No use | 0 – 9 | 10 – 30 | 31+ |  |

Nitrogen dioxides (NO2) and Particulate matter air pollution (PM10) mean exposures.

Due to the presence in UKBB of 4 different years for the concentrations of nitrogen dioxides (2005, 2006, 2007, and 2010), we computed a mean value and used it as a continuous exposure.

As for NO2, we also computed a mean value for PM10 using exposures from the years 2007 and 2010.

**Lden.**

Lden is a measure that represents the overall noise exposure during day, evening, and night periods. To compute this measure, we selected three UKBB exposure that report the average noise due to traffic during daytime (LDay, from 07:00 to 19:00, UKBB data field 24020), evening (LEve, from 19:00 to 23:00, UKBB data field 24021), and night-time (LNight, from 23:00 to 07:00, UKBB data field 24022). Then, the Lden measure was computed as reported by the EU Directive 49/2002 (for the assessment of environmental noise) (<https://eur-lex.europa.eu/legal-content/EN/TXT/PDF/?uri=CELEX:32002L0049&from=EN>), by combining the three average noise measurement and by imposing a penalty for LEve (5dB) and LNight (10dB):

$$L_{den}=10*log(\frac{1}{24}*\left( \left[ 12*\left( {10}^{\frac{L_{day}}{10}} \right) \right]+\left[ 4*\left( {10}^{\frac{L_{evening}+5}{10}} \right) \right]+\left[ 8*\left( {10}^{\frac{L_{night}+10}{10}} \right) \right] \right)$$

**Aspirin, diclofenac, ibuprofen.**

Three non-steroidal anti-inflammatory drugs (NSAIDs) were tested for association with GBM, namely aspirin, diclofenac, and ibuprofen. The use of each one of these NSAIDs was retrieved from the UKBB data field 20003 (“treatment/medication code”), using the following codes: 1140868226 (aspirin), 1140871310 (ibuprofen), and 1140884488 (diclofenac).

**Had sex.**

The “had sex” binary exposure was derived by using the UKBB data field 2139 (“age first had sexual intercourse”). In this latter data field, participants who reported to never had sex (code -2) were coded as 1 in the “had sex” exposure, while all other participants were coded as 0.

**Time spent outdoors.**

The “time spent outdoor” exposure was derived, for each participant, as the mean value of UKBB data field 1050 (“time spend outdoor in summer”) and 1060 (“time spent outdoor in winter”).

**Family history of cancer and family history of depression.**

Family history of cancer and family history of depression were derived from UKBB data fields 20107 (“illnesses of father”) and 20110 (“illnesses of mother”). Both the derived exposures were used as binary exposures.

For family history of cancer, all participants for which a diagnosis of cancer was reported for at least one of the two parents, a value of 1 was assigned, and 0 was assigned otherwise. For family history of cancer, in the UKBB, available diagnoses of cancer are reported for prostate cancer (code 13), breast cancer (code 5), bowel cancer (code 4), and lung cancer (code 3).

For the family history of depression, all participants for which a diagnosis of severe depression (code 12) was reported to at least one of the two parents, a value of 1 was assigned, and 0 was assigned otherwise.

**Weekly usage of mobile phone in last 3 months**

In addition to the derived exposures, the reference category for the “Weekly usage of mobile phone in last 3 months” exposure was also derived from other features in the UKBB cohort. Particularly, the “Weekly usage of mobile phone in last 3 months” feature is comprised of six categories. In this study, individuals who reported never using a mobile phone were selected as the reference category. This category was created by selecting all individuals who indicated that they had never used a mobile phone in the “length of mobile phone use” category (UKBB field 1110).

- 1. **Polygenic score (PGS)**

Full list of the eleven SNPs used to compute the GBM PGS^2^.

| **Chr** | **Pos** | **SNPs** | **A1** | **MAF** | **OR [95% CI]** | **P-value** |
| --- | --- | --- | --- | --- | --- | --- |
| 1 | 64763616 | rs12752552 | T | 0.87 | 1.22 [1.15-1.31] | 2.00E-09 |
| 5 | 1279675 | rs10069690 | T | 0.276 | 1.61 [1.53-1.69] | 8.00E-74 |
| 7 | 54848587 | rs75061358 | G | 0.099 | 1.63 [1.50-1.76] | 5.00E-34 |
| 7 | 55067179 | rs723527 | A | 0.573 | 1.25 [1.20-1.31] | 5.00E-23 |
| 9 | 22032153 | rs634537 | G | 0.411 | 1.37 [1.31-1.43] | 7.00E-45 |
| 11 | 82685972 | rs11233250 | C | 0.868 | 1.24 [1.16-1.33] | 1.00E-09 |
| 16 | 50094961 | rs10852606 | C | 0.713 | 1.18 [1.13-1.24] | 1.00E-11 |
| 16 | 73898 | rs2562152 | T | 0.85 | 1.21 [1.13-1.29] | 2.00E-08 |
| 17 | 7668434 | rs78378222 | G | 0.013 | 2.63 [2.22-3.11] | 5.00E-29 |
| 20 | 63680946 | rs2297440 | C | 0.796 | 1.48 [1.40-1.56] | 4.00E-46 |
| 22 | 38081923 | rs2235573 | G | 0.507 | 1.15 [1.10-1.20] | 2.00E-10 |

The PGS tertiles were computed based on the distribution of the score within the control group, thus resulting in the following categories:

- T1: <18.041
- T2: ≥18.041 and <21.08
- T3 ≥ 21.08

1. **Hyperparameter tuning**

Specifications for the SVM and MLP models are reported below. For each model, the tuned hyperparameters and their respective values is reported. Additionally, the learning curve for each model is also reported. Learning curves are a diagnostic tool used to evaluate the model’s learning performance^3–5^. The training test size (data dimension, X-axis) is plotted against a specific metric of the model (Y-axis). Generally, the closer are the two curves, the better is the generalization ability of a model. In the learning curves reported in this study, each model’s recall (*i.e.,* sensitivity) is reported on the Y-axis and plotted against the data dimension.

- 1. **SVM model**

The following hyperparameters were used to develop the SVM model: *C* = 2.05, *gamma* = 0.001, *kernel* = “rbf”, *class_weight* = “balanced”. The learning curve for the SVM model’s recall is reported in **supplementary figure 1**.

**Supplementary figure 1**. Learning curve for the recall metric of the SVM model.


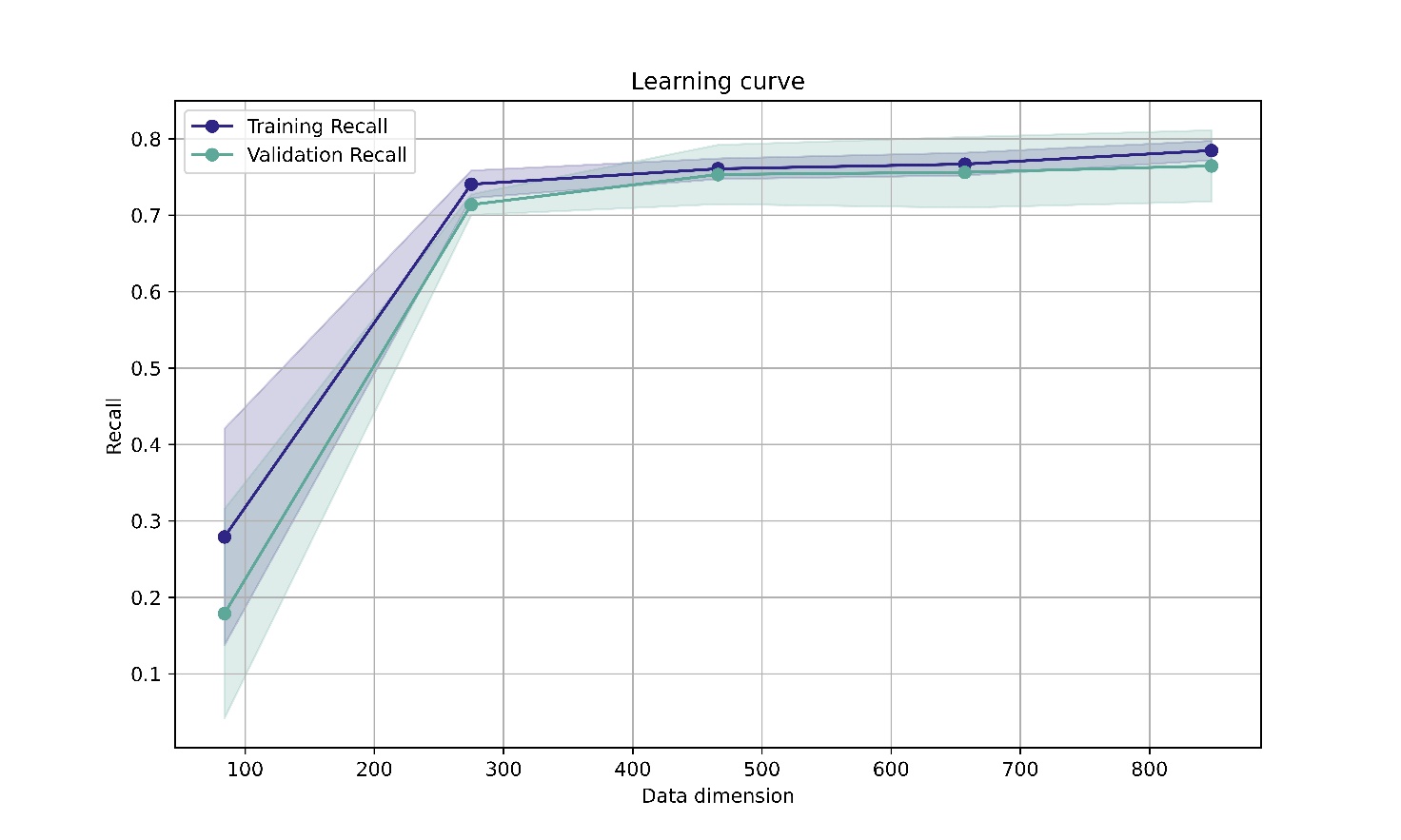


In this learning curve, the recall (*i.e.,* sensitivity) of the Support Vector Machine (SVM) model is plotted against data dimension.

- 1. **MLP model**

The following hyperparameters were used to develop the MLP model: *hidden_layer_sizes* = (12,), *alpha* = 0.002877, *learning_rate_init* = 0.004359, *max_iter* = 823, *warm_start* = TRUE, *solver* = sgd, *random_state* = 1. The learning curve for the MLP model’s recall is reported in **supplementary figure 2**.

**Supplementary figure 2**. Learning curve for the recall metric of the MLP model.


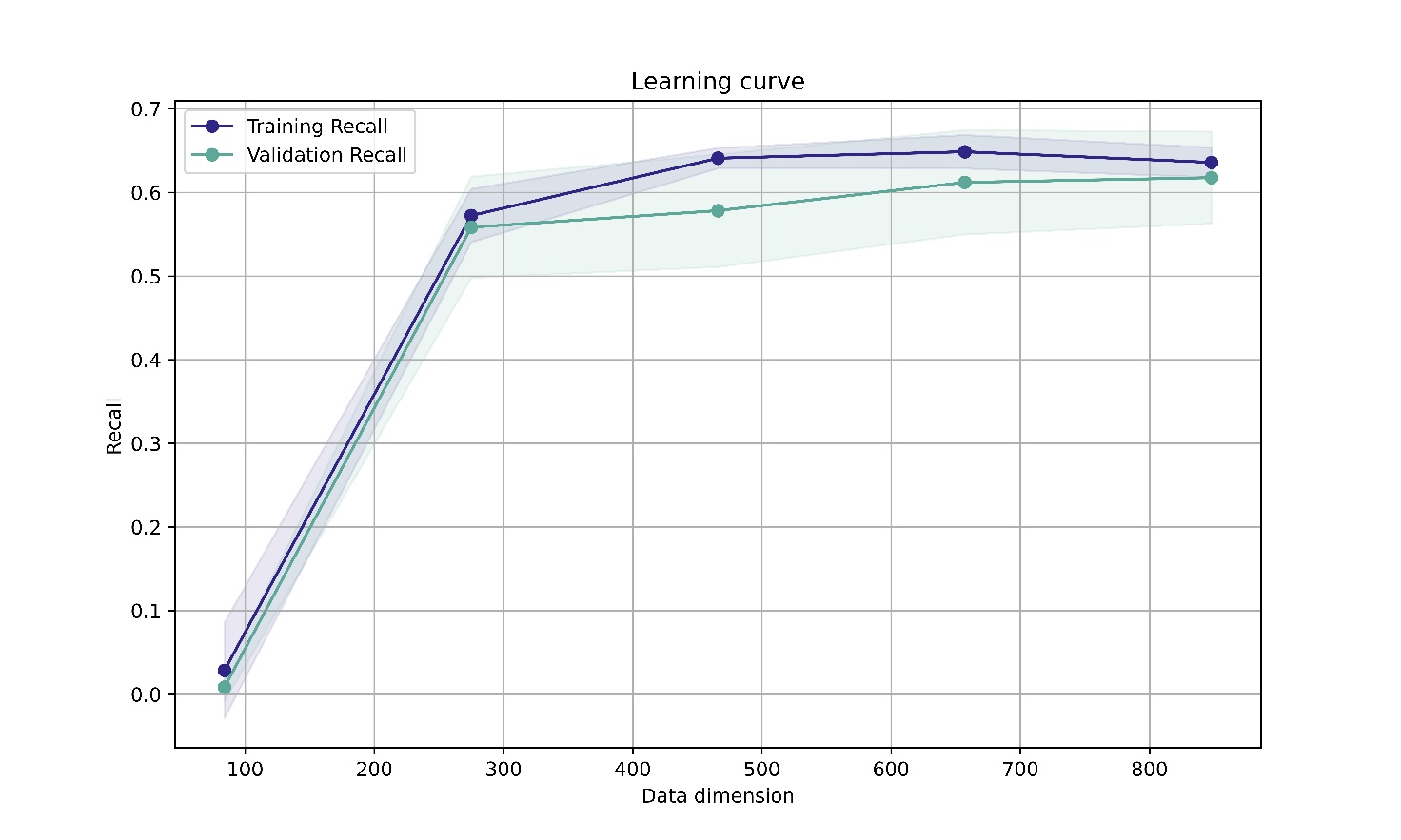


In this learning curve, the recall (*i.e.,* sensitivity) of the Multi-Layer Perceptron (MLP) model is plotted against data dimension.

1. **SHAP Barplots**

**Supplementary figure 3** reports SHAP barplots for the Support Vector Machine (SVM) and the Multi-Layer Perceptron (MLP) models. These plots are used to assess the average impact of a feature on the decisions made by a machine learning model.

**Supplementary figure 3**. SHAP barplots for the SVM and MLP models.


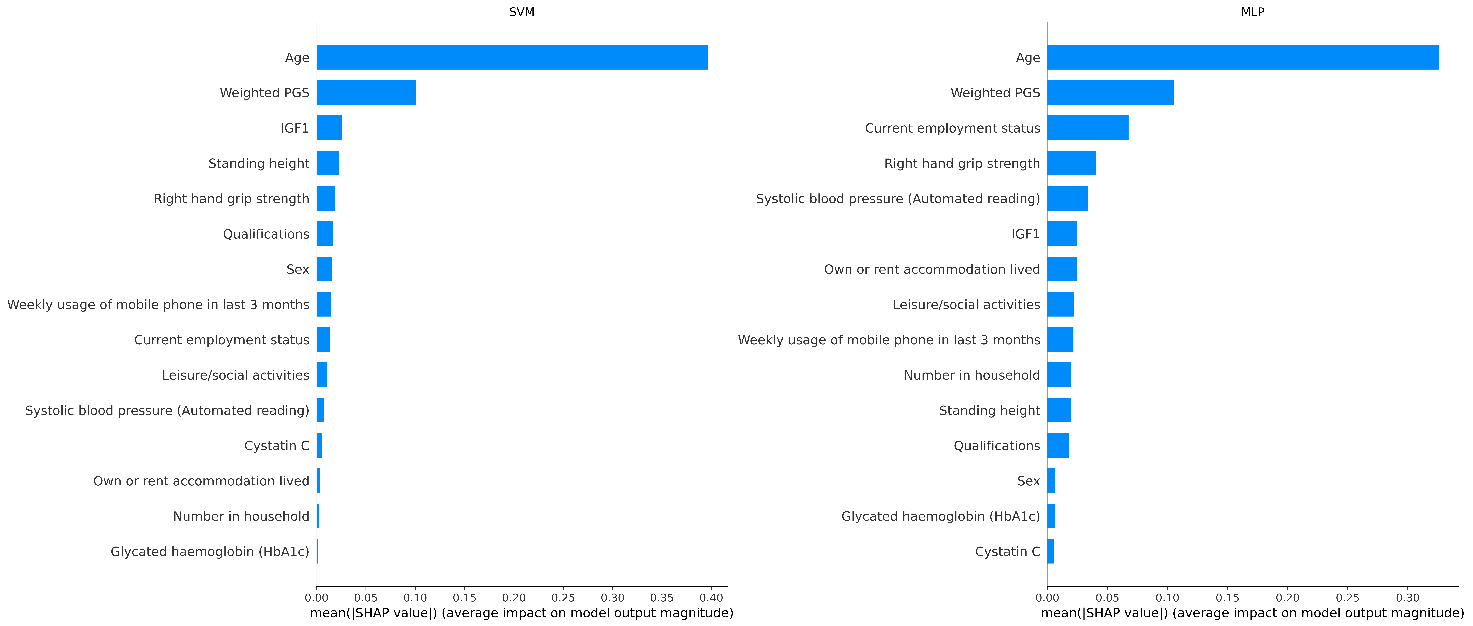


For each feature reported on the Y-axis, the relative impact on each model’s output is reported on the X-axis.

**4. References**

1. Wolpin BM, Kraft P, Gross M, et al. Pancreatic cancer risk and ABO blood group alleles: Results from the Pancreatic Cancer Cohort Consortium. *Cancer Res*. 2010;70(3):1015-1023. doi:10.1158/0008-5472.CAN-09-2993/655687/P/PANCREATIC-CANCER-RISK-AND-ABO-BLOOD-GROUP-ALLELES

2. Melin BS, Barnholtz-Sloan JS, Wrensch MR, et al. Genome-wide association study of glioma subtypes identifies specific differences in genetic susceptibility to glioblastoma and non-glioblastoma tumors. *Nat Genet*. 2017;49(5):789-794. doi:10.1038/ng.3823

3. Viering T, Loog M. The Shape of Learning Curves: a Review. Published online March 19, 2021.

4. Richter AN, Khoshgoftaar TM. Learning Curve Estimation with Large Imbalanced Datasets. In: *2019 18th IEEE International Conference On Machine Learning And Applications (ICMLA)*. IEEE; 2019:763-768. doi:10.1109/ICMLA.2019.00135

5. Mohr F, van Rijn JN. Learning curves for decision making in supervised machine learning: a survey. *Mach Learn*. 2024;113(11-12):8371-8425. doi:10.1007/s10994-024-06619-7
